# Abnormal Functional Connectivity in Cognitive Control Network, Default Mode Network, and Visual Attention Network in Internet Addiction: A Resting-State fMRI Study

**DOI:** 10.3389/fneur.2019.01006

**Published:** 2019-09-18

**Authors:** Yang Wang, Yun Qin, Hui Li, Dezhong Yao, Bo Sun, Zhiliang Li, Xin Li, Yu Dai, Chao Wen, Lingrui Zhang, Chenchen Zhang, Tianmin Zhu, Cheng Luo

**Affiliations:** ^1^School of Acupuncture and Tuina, Chengdu University of Traditional Chinese Medicine, Chengdu, China; ^2^Department of Rehabilitation, Shuangliu Maternal and Child Health Care Hospital, Chengdu, China; ^3^Key Laboratory for NeuroInformation of Ministry of Education, School of Life Science and Technology, University of Electronic Science and Technology of China, Chengdu, China; ^4^School of Medicine, Chengdu University, Chengdu, China; ^5^School of Rehabilitation and Health Preservation, Chengdu University of Traditional Chinese Medicine, Chengdu, China; ^6^Department of Rehabilitation, Zigong Fifth People's Hospital, Zigong, China

**Keywords:** internet addiction, cognitive control network, default mode network, visual attention network, functional connectivity density, substance addiction, attention deficit/hyperactivity disorder

## Abstract

Internet addiction (IA) has become a global mental and social problem, which may lead to a series of psychiatric symptoms including uncontrolled use of internet, and lack of concentration. However, the exact pathophysiology of IA remains unclear. Most of functional connectivity studies were based on pre-selected regions of interest (ROI), which could not provide a comprehensive picture of the communication abnormalities in IA, and might lead to limited or bias observations. Using local functional connectivity density (lFCD), this study aimed to explore the whole-brain abnormalities of functional connectivity in IA. We evaluated the whole-brain lFCD resulting from resting-state fMRI data in 28 IA individuals and 30 demographically matched healthy control subjects (HCs). The correlations between clinical characteristics and aberrant lFCD were also assessed. Compared with HCs, subjects with IA exhibited heightened lFCD values in the right dorsolateral prefrontal cortex (DLPFC), left parahippocampal gyrus (PHG), and cerebellum, and the bilateral middle cingulate cortex (MCC) and superior temporal pole (STP), as well as decreased lFCD values in the right inferior parietal lobe (IPL), and bilateral calcarine and lingual gyrus. Voxel-based correlation analysis revealed the significant correlations between the Young's Internet Addiction Test (IAT) score and altered lFCD values in the left PHG and bilateral STP. These findings revealed the hyper-connectivity in cognitive control network and default mode network as well as the hypo-connectivity in visual attention network, verifying the common mechanism in IA and substance addiction, and the underlying association between IA, and attention deficit/hyperactivity disorder in terms of neurobiology.

## Introduction

Internet addiction (IA), as a behavioral addiction, was conceptualized as the poorly controlled use of internet with impairment of interpersonal relationships, and psychological and social functioning (1). With the popularity of the internet and the improvement of information technology in the last 2 decades, IA has become a serious public health problem due to its high prevalence around the world (2, 3). In Hong Kong, the prevalence rate of IA has dramatic increased from 3.0 to 26.8% in the last 10 years, with the proportion of teenagers who spend more than 20 h online per week increasing from 25.4 to 42.0% (4). The potential risk of IA has attracted growing concerns from worldwide researchers, and mounting studies, and papers have been published. Besides uncontrolled use of internet, multiple comorbid psychiatric symptoms of IA have been revealed by clinical studies, including anxiety, depression, and lack of concentration (5, 6). Epidemiological research further demonstrated that attention deficit/hyperactivity disorder (ADHD), unsociable personality, and bad relationship with parents were high risk factors of IA (6–8). Additionally, ADHD was thought to be the main complication of IA (9). However, the exact pathophysiology of IA has not been fully understood yet, and the underlying mechanism of the coexistence of IA, and comorbid symptoms remains unclear.

Although the pathogenesis has not been well-elucidated, resting-state fMRI provides a useful platform for understanding the neuropathological changes in IA. Resting state functional connectivity (FC), which reflects the temporal coherence of inter-regional spontaneous blood oxygen level dependent (BOLD) fluctuations in cerebral activity (10), was extensively used to investigate the functional interactions between brain areas in psychiatric research, including IA. In previous fMRI studies, IA was associated with the altered FC of cognitive control network (CCN) (11), which was considered as the main neurobiological characteristic of substance addiction. For instance, enhanced FC between the dorsolateral prefrontal cortex (DLPFC), and the temporoparietal junction (TPJ) was observed in IA individuals (12), which was associated with many other cognitive disorders (13, 14). Furthermore, altered FC within the CCN was also demonstrated. Compared with healthy subjects, adolescents with IA exhibited heightened FC between the DLPFC, and anterior cingulate cortex (ACC), correlating with the symptom severity of IA (15). According to Volkow's addiction model, abnormal CCN was proposed as the key determinant in development, and maintenance of substance addiction (16). The altered FC of CCN found in IA individuals, giving a pathophysiological explanation for the uncontrolled use of internet, suggested that IA might share similar neurobiological mechanism with substance addiction (17). However, most previous FC studies in IA were based on a priori selection of region of interest (ROI). This seed-based analysis cannot provide a whole-brain picture of FC alterations in IA, and may lead to limited or biased observations due to the restriction on ROI.

Local functional connectivity density (lFCD) has been developed to analyze the whole-brain FC abnormalities by computing the temporal correlations of every pair of neighboring voxels in the entire brain (18, 19). Voxels with a higher number of functional connections have greater lFCD values, and are considered to be important functional hubs in the information processing. Unlike seed-based FC, lFCD provides an unbiased approach to the study of whole-brain functional connectivity (18). As a result, extra abnormalities have been revealed by lFCD in many disorders, which would not have been detected using traditional seed-based FC analysis methods (20–22). Investigating the alterations of lFCD in IA may provide the full picture of the communication abnormalities of whole-brain functional network, and offer a more reliable, and comprehensive result beyond the FC. This may help to improve our understanding of the neuropathological mechanism of IA and the common neurobiological dysfunction underlying the coexistence of IA, and comorbid symptoms.

In the current study, we aim to investigate the lFCD dysfunction in IA individuals and to detect the potential relationships between the altered lFCD values and the related symptom scores. Local FCD from 28 IA individuals were compared to those of 30 demographically matched healthy controls (HCs). Furthermore, a voxel-based correlation analysis was performed to examine the possible associations between the clinical variables and the lFCD values of the regions with significant differences.

## Materials and Methods

### Participants

A total of 58 subjects (28 IAs and 30 HCs) were recruited from university students. All participants were right-handed and native Chinese speakers. The inclusion criteria for IA group were as follows: (1) aged between 18 and 30 years; (2) in line with the diagnostic criteria of IA via Young's Diagnostic Questionnaire (YDQ) (1); (3) a score of 60 or higher on Young's Internet Addiction Test (IAT); (4) not under any form of therapeutic interventions; and (5) without any other organic or mental diseases. Thirty HCs, well-matched with IA group in age, gender, and education, were recruited with the following inclusion criteria: (1) aged between 18 and 30 years; (2) inconsistent with the IA diagnostic criteria by Young (1); (3) IAT score <50; and (4) without any other organic, or mental diseases. Participants with a history of substance addiction were excluded in our study. Additionally, pregnant, or lactating women were also excluded.

The protocol of this study was evaluated and approved by the Sichuan Regional Ethics Review Committee on traditional Chinese medicine (ethical approval number 2016KL-005), in accordance with the Declaration of Helsinki (2000). All participants signed informed consent forms before inclusion and were compensated for their participation.

### Questionnaire

Besides IAT, the Yale-Brown Obsessive Compulsive Scale (YBOCS) (23) and the Barratt Impulsiveness Scale-11 (BIS-11) (24) were finished by all subjects, and were used to assess obsessive-compulsive and impulsive behaviors, respectively. All scales were translated into Chinese. We also collected information on sex, age, educational level, and years of internet use via self-designed questionnaire.

### Data Acquisition

All fMRI data were collected on a 3.0T MRI scanner (GE Discovery MR 750, USA) with a standard 8-channel head coil. During scanning, participants were instructed to keep their eyes closed and head still, and remain awake without thinking anything in particular. Ear plugs and foam pads were used to restrict noise and displacement of head, respectively. Functional images were acquired using a standard Echo Planar Imaging sequence with the parameters as follows: repetition time = 2000 ms, echo time = 30 ms, flip angle = 90°, field of view = 24 × 24 cm^2^, image matrix = 64 × 64, no gap, and voxel size = 3.75 × 3.75 × 4.4 mm^3^. Two hundred and 55 volumes of image were acquired, and each volume included 35 slices.

### Date Preprocessing

Functional images were preprocessed using the neuroscience information toolbox (NIT, http://www.neuro.uestc.edu.cn/NIT.html), which is based on Statistical Parametric Mapping (SPM8, http://www.fil.ion.ucl.ac.uk/spm/). Prior to preprocessing, the initial 5 volumes of each subject were removed to minimize the interference of instability in the initial signals. Subsequently, slice timing was conducted to correct the time delay between slices. Spatial realignment was performed to correct the head motion. Participants with more than 2 mm displacement or more than 2° rotation were excluded. The fMRI images were then normalized to standard Montreal Neurological Institute (MNI) template with a resolution of 3 × 3 × 3 mm^3^. After that, we regressed out 24 head motion parameters and signals from cerebral spinal fluid, and white matter. Finally, band-pass filtering (0.01–0.08 Hz) was performed to reduce the interference of low-frequency drift and high-frequency noise (25, 26).

### Local FCD Calculation

Local FCD, which is defined as a sum of the number of efficient neighboring functional connections of a given voxel, reflects the voxel-wise whole-brain functional connectivity strength. In our study, lFCD calculation was conducted using the NIT toolbox, based on the proposal by Tomasi and Volkow (18, 19). First, Pearson correlation coefficients between the time course of a given voxel and those of its neighboring voxels were computed. According to the seminal paper (18), functional connections between two voxels with a correlation coefficient *R* > 0.6 were considered significant, as thresholds of *R* < 0.4 might lead to too many false positives, while thresholds of *R* > 0.7 might reduce the sensitivity of lFCD maps (27). All voxels were calculated to generate the whole brain lFCD map. Then we smoothed the lFCD maps with a Gaussian kernel of 6 mm. A more detailed procedure of lFCD calculation can be found in our previous study (28).

### Statistical Analysis

Using SPSS 18.0, we compared demographic data and clinical variables between IA individuals and HCs. Two-sample *t*-test was performed to compare the intergroup differences in continuous variables (e.g., age, years of internet use, scores on clinical scales) while chi-square test was used for categorical variable (e.g., gender). A *P* < 0.05 was considered to be statistically significant. Then a two-sample *t*-test was performed to compare lFCD values between IA individuals and HCs, and a *P* < 0.01 with cluster size > 23 adjacent voxels was deemed significant according to the random field theory (29), which set the threshold for the spatial extent of clusters is extent_−_threshold = 638 mm^3^. In addition, a voxel-based correlation analysis was conducted between the lFCD values and the clinical scores and then the potential association between aberrant lFCD and severity of symptoms was obtained by intersection of lFCD and correlation results. Statistical significance was set at *P* < 0.05 (FDR corrected).

## Results

### Demographic Characteristics and Clinical Measures

Subject demographics and clinical scores of 28 IA individuals and 30 HCs are shown in Table 1. No significant intergroup difference was observed in age, sex, or years of education between two groups (*P* > 0.05). Consistent with the inclusion, IA individuals exhibited higher scores of IAT, YBOCS, BIS-11 significantly (*P* < 0.001), although the years of internet use showed no difference (*P* > 0.05).

**Table 1:** Demographics and clinical characteristics of the individuals with internet addiction and the healthy controls.

	**Internet addiction group (*n* = 28)**	**Healthy control group (*n* = 30)**	***P*-value^#^**
	**m ± sd**	**m ± sd**	
Age (years)	21.32 ± 1.96	21.73 ± 2.08	0.45
Gender (male/female)	21/7	22/8	0.89
Education (years)	15.21 ± 1.84	15.77 ± 1.82	0.26
Internet use (years)	8.14 ± 2.84	8.23 ± 2.33	0.90
Framewise displacement	0.030 ± 0.011	0.031 ± 0.011	0.79
Young's internet addiction test (IAT)	73.89 ± 6.76	29.90 ± 7.18	<0.001
Yale-Brown Obsessive Compulsive Scale (YBOCS)	15.64 ± 7.40	4.30 ± 5.07	<0.001
Barratt Impulsiveness Scale-11 (BIS-11)	79.57 ± 9.37	62.53 ± 8.57	<0.001

# *Two-sample t-test and chi-square test were performed to evaluate the intergroup difference in continuous and categorical variables, respectively*.

### Local FCD Results

**Figure 1:**
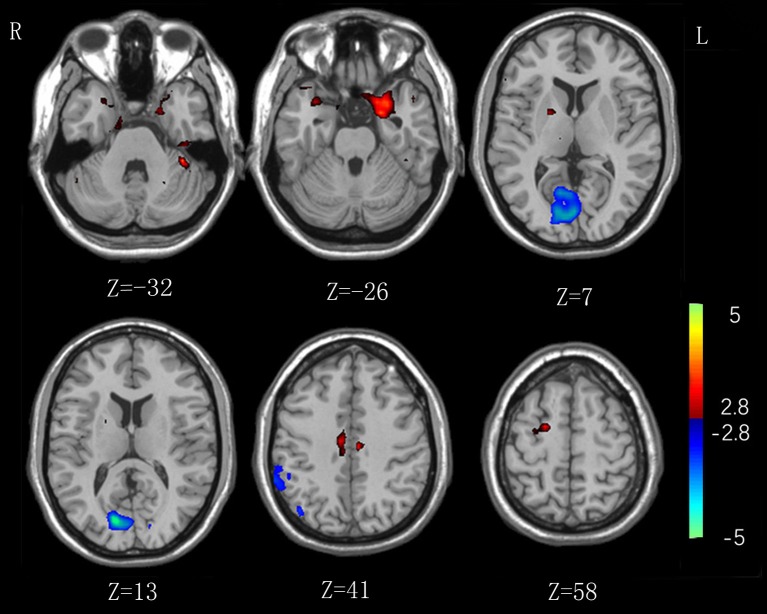
Significant intergroup differences in lFCD between the internet addiction individuals and healthy control subjects. Compared with healthy control subjects, individuals with IA exhibited increased lFCD values in the right superior frontal gyrus, left cerebellum, and parahippocampal gyrus, and bilateral middle cingulum cortex, and superior temporal pole. Decreased lFCD values were exhibited in the right inferior parietal lobe and bilateral calcarine and lingual gyrus.

As shown in Table 2 and Figure 1, in comparison to HCs IA individuals exhibited lower lFCD values in the visual attention regions (*P* < 0.01, with the cluster-level corrected), including the right inferior parietal lobe (IPL, comprising supramarginal gyrus and inferior parietal cortex), and bilateral calcarine and lingual gyrus. Higher lFCD values (*P* < 0.01, with the cluster-level corrected) were observed in brain regions belonging to the DMN, including the left parahippocampal gyrus (PHG), and bilateral superior temporal pole (STP). In addition, enhanced lFCD values in the CCN were also demonstrated (*P* < 0.01, with the cluster-level corrected), including the right DLPFC (comprising superior frontal gyrus), left cerebellum, and bilateral middle cingulum cortex (MCC). We further evaluated the influence of framewise displacement (FD), which might partially index physiological noise (30, 31). No significant intergroup difference in FD and no correlation between FD and lFCD values were found (*P* > 0.05).

**Table 2:** Differences in lFCD between IA individuals and HCs.

**AAL Regions**	**Cluster voxels**	**MNI coordinates**			**Peak *T*-value**	***P* uncorrected**
		**X**	**Y**	**Z**		
CER_L	29	−39	−39	−33	4.2465	<0.01
STP_R	32	9	9	−15	3.8903	<0.01
STP_L	57	−21	10	−30	3.8258	<0.01
PHG_L	40	−21	0	−30	4.1046	<0.01
CAL_R	173	15	−81	9	−5.5509	<0.01
CAL_L	87	3	−85	7	−4.3274	<0.01
LING_R	156	10	−66	4	−4.3057	<0.01
LING_L	53	−6	−82	0	−3.2804	<0.01
SMG_R	42	63	−48	42	−3.6324	<0.01
IPC_R	34	59	−49	42	−3.4958	<0.01
MCC_R	74	9	−15	36	3.7217	<0.01
MCC_L	24	−7	−17	39	3.5067	<0.01
SFG_R	40	21	−3	57	3.6759	<0.01

*lFCD, local functional connectivity density; IA, internet addiction; HCs, healthy controls; MNI, Montreal Neurological Institute; L, left; R, right; CER, cerebellum; STP, superior temporal pole; PHG, parahippocampal gyrus; CAL, calcarine; LING, lingual; SMG, supramarginal gyrus; IPC, inferior parietal cortex; MCC, middle cingulum cortex; SFG, superior frontal gyrus*.

### Relationship Between lFCD Values and Clinical Scores

**Figure 2:**
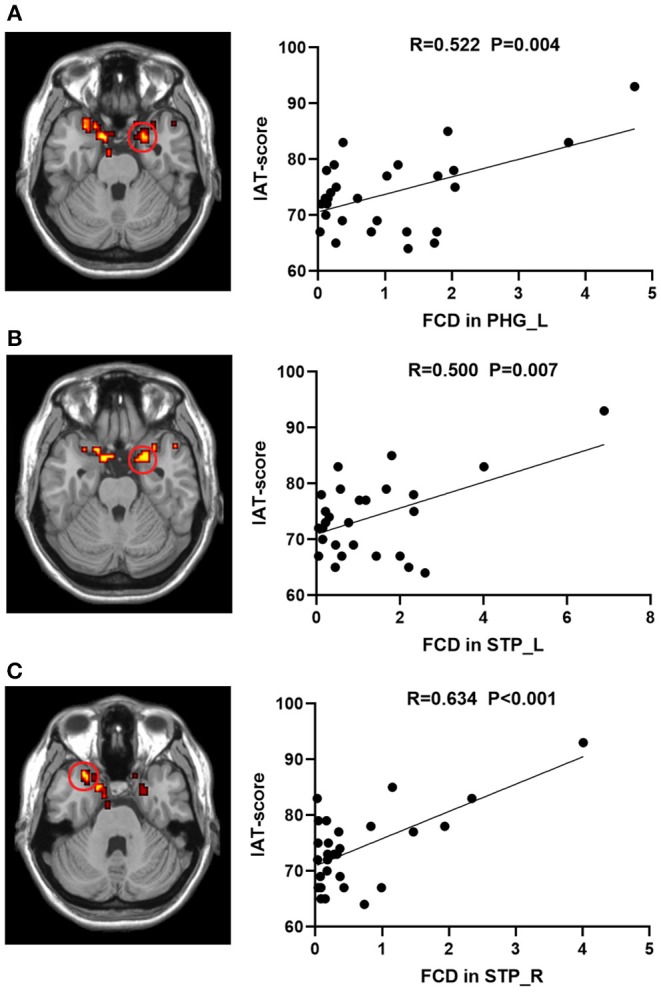
Correlations between lFCD values and IAT score in IA group. The IAT score was positively associated with lFCD values in the **(A)** STP_L, **(B)** STP_R, and **(C)** PHG_L. lFCD, local functional connectivity density; IAT, Young's internet addiction test; L, left; R, right; PHG, parahippocampal gyrus; STP, superior temporal pole.

Voxel-based correlation analysis revealed positive correlations between the IAT score and the heightened lFCD values of left PHG (*r* = 0.522, *P* = 0.004) and bilateral STP (Left: *r* = 0.500, *P* = 0.007; Right: *r* = 0.634, *P* < 0.001) after FDR correction (Figure 2). However, there was no remarkable association between the neural activity changes and BIS-11 or YBOCS after multiple comparison correction, although the IA subjects exhibited higher BIS-11 and YBOCS scores.

## Discussion

Using a voxel-wise data-driven method, the current study investigated the changes of whole-brain functional connectivity in IA individuals. Similar to substance addiction, IA was manifested with enhanced lFCD in the CCN. In addition, increased lFCD in the DMN and decreased lFCD in the visual attention network were both related to impaired attention, and were also observed in our study. Furthermore, we demonstrated the association between the severity of IA and altered lFCD values in DMN. These findings might provide novel insights into the underlying pathophysiological mechanisms of IA and the underlying association between IA and complications.

### Increased lFCD Values in CCN

The CCN, which underlies the ability to allow for appropriate actions through the suppression of inappropriate motor, is thought to be the key determinant in substance dependence model (16). In our study, the IA individuals exhibited heightened lFCD values in the right DLPFC, left cerebellum and bilateral MCC, all of which are main components of the CCN.

The DLPFC is one of the most well-investigated areas in the brain, in particular in the context of cognitive function. As the core node of CCN, the DLPFC is involved in a variety of advanced cognitive functions essential for decision-making and goal-directed behavior, such as self-control (32), action planning (33), and intertemporal choice (34). Dysfunction in DLPFC has been found in many cognitive control disorders (35–37), in particular in substance dependence (38, 39). Evidence from previous neuroimaging studies has demonstrated the pivotal role of DLPFC dysfunction in craving for marijuana (40), cigarette (41), and alcohol (42), and, thus, the altered DLPFC function was presumed as a neuropathological explanation of substance addiction (43). The cingulate cortex, involved in cognition and decision making, is also implicated in various addictions (44, 45). As a vital component of cingulate cortex, the MCC is engaged in decision making for reward/approach and fear/avoidance selection by assessment of potential outcomes (46, 47), and therefore closely related to substance dependence (48).

As to IA, multiple studies attempting to elucidate the pathological mechanism of IA have converged on the abnormalities of the DLPFC and cingulate cortex (49, 50). Higher Regional homogeneity (ReHo) values in the DLPFC and cingulate cortex have been revealed, reflecting the enhanced synchronization between the DLPFC, and cingulate cortex in individuals with IA (51). Additionally, hyperactivity in the DLPFC and cingulate cortex was also found when the IA subjects were exposed to an internet-related cue (52). Furthermore, altered gray matter in the DLPFC and MCC were revealed in IA group by structural MRI (sMRI), suggesting that the functional alterations in the DLPFC, and MCC were based on the structural abnormalities (53, 54). In our study, the heightened lFCD values in the right DLPFC and bilateral MCC observed in IA individuals further demonstrated the dysfunctional CCN in IA, providing new evidence supporting the hypothesis that IA shares similar neurobiological mechanism with substance addiction.

The cerebellum was traditionally considered as a motor center, well-known for its involvement in postural, and motor control. However, in recent studies, cerebellum abnormalities were found in cognitive disorders (55, 56). Conversely, cerebellum resections or lesions caused various types of abnormal social behavior (57, 58). Providing new insights into the cerebellum function, recent studies revealed the role of cerebellum in cognitive control function (59, 60). Furthermore, structural and functional changes in the cerebellum were found in substance-dependent individuals who exhibited uncontrolled craving for substance (61, 62). Similar to those in substance addiction, cerebellum abnormalities were also demonstrated in IA, associated with the severity of addiction (63). Consistent with the previous studies, our results proved the remarkably increased lFCD in cerebellum in IA group, supporting the speculation that there were homological neurobiological changes in the CCN in IA and substance addiction.

### Increased lFCD in DMN and Decreased lFCD in Visual Attention Network

The DMN is generally regarded as the most significant component of the resting state network, due to its typical deactivation during attention-demanding cognitive tasks and activation during internal mentation (e.g., retrieval of episodic memory, self-referential, and imagination of future) (64–67). Dysfunctional DMN is implicated in numerous mental disorders, in particular in ADHD. In our study, enhanced lFCD values in the left PHG and bilateral STP, both are integral parts of the DMN, were revealed in IA group.

As an important part of limbic system, the PHG is responding to emotional, sensory, and environmental stimuli (68–70), and involved in contextual associations, which means processing schema (object-context) associations (71). Due to the function of linking specific sensations to contextual predictions, the PHG is thought to play an essential role in attention (72). Similarly, as an integral component of paralimbic region, the temporal pole is involved in spatial navigation and retrieval of memory (73, 74), which are also thought to be important in attentional allocation (72). In previous studies, structural, and functional alterations in the PHG and temporal pole were implicated in the neuropathology of ADHD (75–78). Consistent with those in ADHD, lFCD in the PHG and STP were revealed in our study, implicating the enhanced functional connectivity of DMN in IA individuals.

Contrary to the enhanced DMN, the weaker lFCD values in the right IPL, and bilateral calcarine, and lingual gyrus were observed in our study. All of these regions are components of the visual attention network (79, 80), involved in the bottom-up attentional pathway which is proposed as a circuit-breaker to reorient attention to new and external information (81). Abnormalities in visual attention network were associated with impaired concentration in previous studies (72, 82).

As the primary visual cortex, the calcarine plays a major role in visual information integration and attention processing (83). Structural and functional alterations in the calcarine were revealed in ADHD (84, 85). As the secondary visual cortex, the lingual gyrus has a critical function in spatial memory (86), and visual attention (87). Likewise, abnormalities in the lingual gyrus in ADHD children were identified in the existing literatures (88, 89). Due to its core role in attentional reorienting and visuomotor integration (80), the IPL is regarded as a supramodal core in the ventral attention network. Hypoactivity in ventral attention network, implicating insensitivity with external environment, is proposed as a key determinant in impairment of attention (90). Both structural and functional abnormalities in IPL were associated with ADHD in previous studies (91, 92). As to IA, abnormalities in the IPL and lingual gyrus were also revealed in IA adolescents, implicating the role of visual attention network in the impaired concentration in IA (93, 94).

The association between IA and ADHD has been demonstrated in previous epidemiology studies. ADHD is considered as the most remarkably associated mental disorder of IA (9). Adolescents with IA have obviously higher ADHD symptoms, among which lack of concentration is the most associated one with IA (95). Prior neural evidence has implicated the importance of hyper-connectivity within DMN (96, 97) and hypo-connectivity within the visual attention network (98) in the inattention in patients with ADHD. In our study, the increased lFCD values in the left PHG and bilateral STP and the decreased lFCD values in the right IPL and bilateral calcarine and lingual gyrus were observed, indicating the heightened functional connectivity of DMN and reduced functional connectivity of visual attention network in IA, which were similar with those in ADHD. To our knowledge, this was the first time the STP and calcarine were associated to IA. These findings, providing direct neural explanation for the impaired concentration in IA, demonstrated the underlying association between IA and ADHD in terms of neurobiology. Additionally, we further revealed the positive correlations between the IAT score and the lFCD in left PHG and bilateral STP, implicating the pivotal role of DMN dysfunction in development of IA.

### Limitations

Our study has several limitations. First, FCD was restricted to the local functional connectivity in our study. We did not calculate the global FCD due to the misgiving of its high variability (99). And furthermore, according to the seminal paper, the local FCD, and global FCD are proportional to one another (18). Second, due to the small subject size and the sex difference in morbidity, our study fails to analyze the intergender differences. Finally, subjects are restricted to college students, which limits the generalization of this study.

## Conclusion

In the present study, a voxel-wise method was performed to analyze the whole-brain FC abnormalities in IA subjects. Our results revealed that individuals with IA exhibited heightened lFCD values in the CCN and DMN as well as decreased lFCD values in the visual attention network. Our observations, providing neural explanation for the lack of concentration and uncontrolled use of internet in IA individuals, verified the similar neurobiological mechanism in IA with that in substance addiction, and implicated the underlying association between IA and ADHD in terms of neurobiology.

## Data Availability

The datasets generated for this study are available on request to the corresponding author.

## Ethics Statement

This study was carried out in accordance with the recommendations of Ethical Principles for Medical Research Involving Human of Chengdu University of TCM with written informed consent from all subjects. All subjects gave written informed consent in accordance with the Declaration of Helsinki. The protocol was approved by the Sichuan Regional Ethics Review Committee on traditional Chinese medicine.

## Author Contributions

TZ, CL, and DY conceptualized the study, designed the protocol, and administered the project. HL supervised the study. YW, YD, CW, LZ, and CZ preformed the experiments. YQ, BS, ZL, and XL conducted the statistical analysis. YW wrote the first draft of the manuscript. All authors read and approved the manuscript.

### Conflict of Interest Statement

The authors declare that the research was conducted in the absence of any commercial or financial relationships that could be construed as a potential conflict of interest.

## References

[1] Young KS Internet addiction: the emergence of a new clinical disorder. CyberPsychol Behav. (1998) 1:237–44. 10.1089/cpb.1998.1.237

[2] Sevelko K Bischof G Bischof A Besser B John U Meyer C. The role of self-esteem in Internet addiction within the context of comorbid mental disorders: findings from a general population-based sample. J Behav Addict. (2018) 7:976–84. 10.1556/2006.7.2018.130 30585501 PMC6376382

[3] Li L Xu DD Chai JX Wang D Li L Zhang L. Prevalence of Internet addiction disorder in Chinese university students: a comprehensive meta-analysis of observational studies. J Behav Addict. (2018) 7:610–23. 10.1556/2006.7.2018.53 30010411 PMC6426360

[4] Chung TWH Sum SMY Chan MWL. Adolescent internet addiction in hong kong: prevalence, psychosocial correlates, and prevention. J Adoles Health. (2019) 64:S34–43. 10.1016/j.jadohealth.2018.12.016 31122547

[5] Weinstein A Lejoyeux M Internet addiction or excessive internet use. Am J Drug Alcohol Abuse. (2010) 36:277–83. 10.3109/00952990.2010.491880 20545603

[6] Lam LT. Risk factors of Internet addiction and the health effect of internet addiction on adolescents: a systematic review of longitudinal and prospective studies. Curr Psychiatry Rep. (2014) 16:508. 10.1007/s11920-014-0508-2 25212714

[7] Trumello C Babore A Candelori C Morelli M Bianchi D. Relationship with parents, emotion regulation, and callous-unemotional traits in adolescents' internet addiction. Biomed Res Int. (2018) 2018:7914261. 10.1155/2018/7914261 29951544 PMC5989287

[8] Chou WJ Liu TL Yang P Yen CF Hu HF. Multi-dimensional correlates of Internet addiction symptoms in adolescents with attention-deficit/hyperactivity disorder. Psychiatry Res. (2015) 225:122–8. 10.1016/j.psychres.2014.11.003 25466226

[9] Ko CH Yen JY Chen CS Chen CC Yen CF. Psychiatric comorbidity of internet addiction in college students: an interview study. CNS Spectr. (2008) 13:147–53. 10.1017/S1092852900016308 18227746

[10] Biswal B Yetkin FZ Haughton VM Hyde JS. Functional connectivity in the motor cortex of resting human brain using echo-planar MRI. Magnetic Res Med. (1995) 34:537–41. 10.1002/mrm.1910340409 8524021

[11] Weinstein AM. An update overview on brain imaging studies of internet gaming disorder. Front Psychiatry. (2017) 8:185. 10.3389/fpsyt.2017.00185 29033857 PMC5626837

[12] Han DH Kim SM Bae S Renshaw PF Anderson JS. Brain connectivity and psychiatric comorbidity in adolescents with Internet gaming disorder. Addict Biol. (2017) 22:802–12. 10.1111/adb.12347 26689148

[13] Anderson JS Nielsen JA Froehlich AL DuBray MB Druzgal TJ Cariello AN. Functional connectivity magnetic resonance imaging classification of autism. Brain. (2011) 134:3739–51. 10.1093/brain/awr263 22006979 PMC3235557

[14] Anderson JS Nielsen JA Ferguson MA Burback MC Cox ET Dai L. Abnormal brain synchrony in down syndrome. Neuroimage Clinical. (2013) 2:703–15. 10.1016/j.nicl.2013.05.006 24179822 PMC3778249

[15] Li W Li Y Yang W Zhang Q Wei D Li W. Brain structures and functional connectivity associated with individual differences in Internet tendency in healthy young adults. Neuropsychologia. (2015) 70:134–44. 10.1016/j.neuropsychologia.2015.02.019 25698637

[16] Volkow ND Wang GJ Fowler JS Tomasi D Telang F Baler R. Addiction: decreased reward sensitivity and increased expectation sensitivity conspire to overwhelm the brain's control circuit. Bioessays. (2010) 32:748–55. 10.1002/bies.201000042 20730946 PMC2948245

[17] Kim HS Hodgins DC. Component model of addiction treatment: a pragmatic transdiagnostic treatment model of behavioral and substance addictions. Front Psychiatry. (2018) 9:406. 10.3389/fpsyt.2018.00406 30233427 PMC6127248

[18] Tomasi D Volkow ND. Functional connectivity density mapping. Proc Natl Acad Sci USA. (2010) 107:9885–90. 10.1073/pnas.1001414107 20457896 PMC2906909

[19] Tomasi D Volkow ND. Ultrafast method for mapping local functional connectivity hubs in the human brain. Conf Proc IEEE Eng Med Biol Soc. (2010) 2010:4274–7. 10.1109/IEMBS.2010.5626180 21095749 PMC3184891

[20] Qin W Xuan Y Liu Y Jiang T Yu C. Functional connectivity density in congenitally and late blind subjects. Cereb Cortex. (2015) 25:2507–16. 10.1093/cercor/bhu051 24642421

[21] Manza P Tomasi D Volkow ND. Subcortical local functional hyperconnectivity in cannabis dependence. Biol Psychiatry. (2018) 3:285–93. 10.1016/j.bpsc.2017.11.004 29486870 PMC5833305

[22] Shokri-Kojori E Tomasi D Wiers CE Wang GJ Volkow ND. Alcohol affects brain functional connectivity and its coupling with behavior: greater effects in male heavy drinkers. Mol Psychiatry. (2017) 22:1185–95. 10.1038/mp.2016.25 27021821 PMC5138152

[23] Goodman WK Price LH Rasmussen SA Mazure C Delgado P Heninger GR The yale-brown obsessive compulsive scale. II. validity. Arch Gen Psychiatry. (1989) 46:1012–6. 10.1001/archpsyc.1989.01810110054008 2510699

[24] Patton JH Stanford MS Barratt ES. Factor structure of the Barratt impulsiveness scale. J Clin Psychol. (1995) 51:768–74. 10.1002/1097-4679(199511)51:6<768::AID-JCLP2270510607>3.0.CO;2-1 8778124

[25] Cordes D Haughton VM Arfanakis K Carew JD Turski PA Moritz CH. Frequencies contributing to functional connectivity in the cerebral cortex in resting-state data. AJNR Am. J. Neuroradiol. (2001) 22:1326–33. 11498421 PMC7975218

[26] Liu T Li J Zhang Z Xu Q Lu G Huang S. Altered long- and short-range functional connectivity in patients with betel quid dependence: a resting-state functional MRI study. Cell Physiol Biochem. (2016) 40:1626–36. 10.1159/000453212 28006783

[27] Cohen AD Tomasi D Shokri-Kojori E Nencka AS Wang Y. Functional connectivity density mapping: comparing multiband and conventional EPI protocols. Brain Imaging Behav. (2018) 12:848–59. 10.1007/s11682-017-9742-7 28676985

[28] Luo C Tu SP Peng YH Gao S Li JF Dong L. Long-term effects of musical training and functional plasticity in salience system. Neural Plast. (2014) 2014:180138. 10.1155/2014/180138 25478236 PMC4247966

[29] Friston KJ. Testing for anatomically specified regional effects. Hum Brain Mapp. (1997) 5:133–6. 10.1002/(SICI)1097-0193(1997)5:2<133::AID-HBM7>3.0.CO;2-4 10096418

[30] Power JD. A simple but useful way to assess fMRI scan qualities. NeuroImage. (2017) 154:150–8. 10.1016/j.neuroimage.2016.08.009 27510328 PMC5296400

[31] Power JD Plitt M Laumann TO Martin A. Sources and implications of whole-brain fMRI signals in humans. NeuroImage. (2017) 146:609–25. 10.1016/j.neuroimage.2016.09.038 27751941 PMC5321814

[32] Hare TA Camerer CF Rangel A. Self-control in decision-making involves modulation of the vmPFC valuation system. Science. (2009) 324:646–8. 10.1126/science.1168450 19407204

[33] Mushiake H Saito N Sakamoto K Itoyama Y Tanji J. Activity in the lateral prefrontal cortex reflects multiple steps of future events in action plans. Neuron. (2006) 50:631–41. 10.1016/j.neuron.2006.03.045 16701212

[34] Kim S Hwang J Lee D. Prefrontal coding of temporally discounted values during intertemporal choice. Neuron. (2008) 59:161–72. 10.1016/j.neuron.2008.05.010 18614037 PMC2593737

[35] Hoppenbrouwers SS De DR Stirpe T Fitzgerald PB Voineskos AN Schutter D. Inhibitory deficits in the dorsolateral prefrontal cortex in psychopathic offenders. Cortex. (2013) 49:1377–85. 10.1016/j.cortex.2012.06.003 22795183

[36] Weygandt M Mai K Dommes E Leupelt V Hackmack K Kahnt T. The role of neural impulse control mechanisms for dietary success in obesity. Neuroimage. (2013) 83:669–78. 10.1016/j.neuroimage.2013.07.028 23867558

[37] Weygandt M Mai K Dommes E Ritter K Leupelt V Spranger J. Impulse control in the dorsolateral prefrontal cortex counteracts post-diet weight regain in obesity. Neuroimage. (2015) 109:318–27. 10.1016/j.neuroimage.2014.12.073 25576647

[38] Ernst LH Plichta MM Dresler T Zesewitz AK Tupak SV Haeussinger FB. Prefrontal correlates of approach preferences for alcohol stimuli in alcohol dependence. Addict Biol. (2014) 19:497–508. 10.1111/adb.12005 23145772

[39] Chang D Zhang J Peng W Shen ZW Gao X Du YH. Smoking cessation with 20 Hz repetitive transcranial magnetic stimulation (rTMS) applied to two brain regions: a pilot study. Front Human Neurosci. (2018) 12:344. 10.3389/fnhum.2018.00344 30319373 PMC6166007

[40] Boggio PS Zaghi S Villani AB Fecteau S Pascual-Leone A Fregni F. Modulation of risk-taking in marijuana users by transcranial direct current stimulation (tDCS) of the dorsolateral prefrontal cortex (DLPFC). Drug Alcohol Depend. (2010) 112:220–5. 10.1016/j.drugalcdep.2010.06.019 20729009

[41] Hayashi T Ko JH Strafella AP Dagher A. Dorsolateral prefrontal and orbitofrontal cortex interactions during self-control of cigarette craving. Proc Natl Acad Sci USA. (2013) 110:4422–7. 10.1073/pnas.1212185110 23359677 PMC3600476

[42] Klauss J Pinheiro LCP Merlo BLS Santos GDC Fregni F Nitsche MA. A randomized controlled trial of targeted prefrontal cortex modulation with tDCS in patients with alcohol dependence. Int J Neuropsychopharmacol. (2014) 17:1793–803. 10.1017/S1461145714000984 25008145

[43] Owens MM Syan SK Amlung M Beach SRH Sweet LH MacKillop J. Functional and structural neuroimaging studies of delayed reward discounting in addiction: a systematic review. Psychol Bull. (2019) 145:141–64. 10.1037/bul0000181 30652907

[44] Ersche KD Barnes A Jones PS Morein-Zamir S Robbins TW Bullmore ET. Abnormal structure of frontostriatal brain systems is associated with aspects of impulsivity and compulsivity in cocaine dependence. Brain. (2011) 134:2013–24. 10.1093/brain/awr138 21690575 PMC3122375

[45] Schacht JP Anton RF Myrick H. Functional neuroimaging studies of alcohol cue reactivity: a quantitative meta-analysis and systematic review. Addict Biol. (2013) 18:121–33. 10.1111/j.1369-1600.2012.00464.x 22574861 PMC3419322

[46] Vogt BA. Submodalities of emotion in the context of cingulate subregions. Cortex. (2014) 59:197–202. 10.1016/j.cortex.2014.04.002 24933713

[47] Vogt BA. Midcingulate cortex: structure, connections, homologies, functions and diseases. J Chem Neuroanat. (2016) 74:28–46. 10.1016/j.jchemneu.2016.01.010 26993424

[48] Kuhn J Grundler TO Bauer R Huff W Fischer AG Lenartz D. Successful deep brain stimulation of the nucleus accumbens in severe alcohol dependence is associated with changed performance monitoring. Addict Biol. (2011) 16:620–3. 10.1111/j.1369-1600.2011.00337.x 21762290

[49] Du X Yang Y Gao P Qi X Du G Zhang Y. Compensatory increase of functional connectivity density in adolescents with internet gaming disorder. Brain Imaging Behav. (2017) 11:1901–9. 10.1007/s11682-016-9655-x 27975158

[50] Chun J-W Choi J Cho H Choi M-R Ahn K-J Choi J-S. Role of frontostriatal connectivity in adolescents with excessive smartphone use. Front Psychiatry. (2018) 9:437. 10.3389/fpsyt.2018.00437 30258373 PMC6143708

[51] Liu J Gao XP Osunde I Li X Zhou SK Zheng HR. Increased regional homogeneity in internet addiction disorder: a resting state functional magnetic resonance imaging study. Chinese Med J. (2010) 123:1904–8. 10.3760/cma.j.issn.0366-6999.2010.14.014 20819576

[52] Ko CH Liu GC Yen JY Chen CY Yen CF Chen CS. Brain correlates of craving for online gaming under cue exposure in subjects with internet gaming addiction and in remitted subjects. Addict Biol. (2013) 18:559–69. 10.1111/j.1369-1600.2011.00405.x 22026537

[53] Jin CW Zhang T Cai CX Bi YZ Li YD Yu DH. Abnormal prefrontal cortex resting state functional connectivity and severity of internet gaming disorder. Brain Imaging Behav. (2016) 10:719–29. 10.1007/s11682-015-9439-8 26311395

[54] He QH Turel O Bechara A. Brain anatomy alterations associated with social networking site (SNS) addiction. Sci Rep. (2017) 7:8. 10.1038/srep45064 28332625 PMC5362930

[55] Zarei M Mataix-Cols D Heyman I Hough M Doherty J Burge L. Changes in gray matter volume and white matter microstructure in adolescents with obsessive-compulsive disorder. Biol Psychiatry. (2011) 70:1083–90. 10.1016/j.biopsych.2011.06.032 21903200

[56] Seidman LJ Biederman J Liang L Valera EM Monuteaux MC Brown A. Gray matter alterations in adults with attention-deficit/hyperactivity disorder identified by voxel based morphometry. Biol Psychiatry. (2011) 69:857–66. 10.1016/j.biopsych.2010.09.053 21183160 PMC3940267

[57] Hoche F Guell X Sherman JC Vangel MG Schmahmann JD. Cerebellar contribution to social cognition. Cerebellum. (2016) 15:732–43. 10.1007/s12311-015-0746-9 26585120 PMC5157127

[58] Schmahmann JD Sherman JC. The cerebellar cognitive affective syndrome. Brain. (1998) 121(Pt 4):561–79. 10.1093/brain/121.4.561 9577385

[59] Carta I Chen CH Schott AL Dorizan S Khodakhah K. Cerebellar modulation of the reward circuitry and social behavior. Science. (2019) 363:eaav0581. 10.1126/science.aav0581 30655412 PMC6711161

[60] Caulfield MD Zhu DC McAuley JD Servatius RJ. Individual differences in resting-state functional connectivity with the executive network: support for a cerebellar role in anxiety vulnerability. Brain Struct Funct. (2016) 221:3081–93. 10.1007/s00429-015-1088-6 26231515

[61] Hester R Garavan H. Executive dysfunction in cocaine addiction: evidence for discordant frontal, cingulate, and cerebellar activity. J Neurosci. (2004) 24:11017–22. 10.1523/JNEUROSCI.3321-04.2004 15590917 PMC6730277

[62] Brody AL Mandelkern MA Jarvik ME Lee GS Smith EC Huang JC. Differences between smokers and nonsmokers in regional gray matter volumes and densities. Biol Psychiatry. (2004) 55:77–84. 10.1016/S0006-3223(03)00610-3 14706428

[63] Ding WN Sun JH Sun YW Zhou Y Li L Xu JR. Altered default network resting-state functional connectivity in adolescents with Internet gaming addiction. PLoS ONE. (2013) 8:e59902. 10.1371/journal.pone.0059902 23555827 PMC3608539

[64] Schacter DL Addis DR Buckner RL. Remembering the past to imagine the future: the prospective brain. Nat Rev Neurosci. (2007) 8:657–61. 10.1038/nrn2213 17700624

[65] Spreng RN Mar RA Kim AS. The common neural basis of autobiographical memory, prospection, navigation, theory of mind, and the default mode: a quantitative meta-analysis. J Cogn Neurosci. (2009) 21:489–510. 10.1162/jocn.2008.21029 18510452

[66] Amodio DM Frith CD. Meeting of minds: the medial frontal cortex and social cognition. Nat Rev Neurosci. (2006) 7:268–77. 10.1038/nrn1884 16552413

[67] Simpson JR Jr Drevets WC Snyder AZ Gusnard DA Raichle ME. Emotion-induced changes in human medial prefrontal cortex: II. During anticipatory anxiety. Proc Natl Acad Sci USA. (2001) 98:688–93. 10.1073/pnas.98.2.688 11209066 PMC14649

[68] Van den Stock J Vandenbulcke M Sinke CB de Gelder B Affective scenes influence fear perception of individual body expressions. Hum Brain Mapp. (2014) 35:492–502. 10.1002/hbm.22195 23097235 PMC6869608

[69] Rajimehr R Devaney KJ Bilenko NY Young JC Tootell RB. The parahippocampal place area responds preferentially to high spatial frequencies in humans and monkeys. PLoS Biol. (2011) 9:e1000608. 10.1371/journal.pbio.1000608 21483719 PMC3071373

[70] Levy I Hasson U Avidan G Hendler T Malach R. Center-periphery organization of human object areas. Nat Neurosci. (2001) 4:533–9. 10.1038/87490 11319563

[71] Aminoff EM Kveraga K Bar M. The role of the parahippocampal cortex in cognition. Trends Cogn Sci. (2013) 17:379–90. 10.1016/j.tics.2013.06.009 23850264 PMC3786097

[72] Ramzaoui H Faure S Spotorno S. Alzheimer's Disease, visual search, and instrumental activities of daily living: a review and a new perspective on attention and eye movements. J Alzheimers Dis. (2018) 66:901–25. 10.3233/JAD-180043 30400086

[73] Frith U Frith CD. Development and neurophysiology of mentalizing. Philos Trans R Soc Lond B Biol Sci. (2003) 358:459–73. 10.1098/rstb.2002.1218 12689373 PMC1693139

[74] Shikauchi Y Ishii S. Robust encoding of scene anticipation during human spatial navigation. Sci Rep. (2016) 6:14. 10.1038/srep37599 27874089 PMC5118749

[75] Fernandez-Jaen A Lopez-Martin S Albert J Fernandez-Mayoralas DM Fernandez-Perrone AL Tapia DQ. Cortical thinning of temporal pole and orbitofrontal cortex in medication-naive children and adolescents with ADHD. Psychiatry Res Neuroimaging. (2014) 224:8–13. 10.1016/j.pscychresns.2014.07.004 25085707

[76] Van Dessel J Sonuga-Barke E Moerkerke M Van der Oord S Lemiere J Morsink S. The amygdala in adolescents with attention-deficit/hyperactivity disorder: structural and functional correlates of delay aversion. World J Biol Psychiatry. (2019). 10.1080/15622975.2019.1585946. [Epub ahead of print]. 30945592

[77] Wilbertz G Delgado MR Van Elst LT Maier S Philipsen A Blechert J. Neural response during anticipation of monetary loss is elevated in adult attention deficit hyperactivity disorder. World J Biol Psychiatry. (2017) 18:268–78. 10.3109/15622975.2015.1112032 26508322

[78] Kim JH Chung YI Lee JS Kim IJ Kim YK Kim SJ Voxel-based statistical analysis of regional cerebral glucose metabolism in children with attention-deficit hyperactivity disorder. Neural Regenerat Res. (2011) 6:2850–5. 10.3969/j.issn.1673-5374.2011.36.009

[79] Goodale MA Milner AD. Separate visual pathways for perception and action. Trends Neurosci. (1992) 15:20–5. 10.1016/0166-2236(92)90344-8 1374953

[80] Igelstrom KM Graziano MSA. The inferior parietal lobule and temporoparietal junction: a network perspective. Neuropsychologia. (2017) 105:70–83. 10.1016/j.neuropsychologia.2017.01.001 28057458

[81] Corbetta M Patel G Shulman GL. The reorienting system of the human brain: from environment to theory of mind. Neuron. (2008) 58:306–24. 10.1016/j.neuron.2008.04.017 18466742 PMC2441869

[82] Eggebrecht AT Elison JT Feczko E Todorov A Wolff JJ Kandala S. Joint attention and brain functional connectivity in infants and toddlers. Cereb Cortex. (2017) 27:1709–20. 10.1093/cercor/bhw403 28062515 PMC5452276

[83] Bezdek MA Gerrig RJ Wenzel WG Shin J Pirog Revill K Schumacher EH. Neural evidence that suspense narrows attentional focus. Neuroscience. (2015) 303:338–45. 10.1016/j.neuroscience.2015.06.055 26143014

[84] Kumar U Arya A Agarwal V. Neural alterations in ADHD children as indicated by voxel-based cortical thickness and morphometry analysis. Brain Dev. (2017) 39:403–10. 10.1016/j.braindev.2016.12.002 28057397

[85] Saad JF Griffiths KR Kohn MR Clarke S Williams LM Korgaonkar MS. Regional brain network organization distinguishes the combined and inattentive subtypes of attention deficit hyperactivity disorder. Neuroimage Clin. (2017) 15:383–90. 10.1016/j.nicl.2017.05.016 28580295 PMC5447655

[86] Sulpizio V Committeri G Lambrey S Berthoz A Galati G. Selective role of lingual/parahippocampal gyrus and retrosplenial complex in spatial memory across viewpoint changes relative to the environmental reference frame. Behav Brain Res. (2013) 242:62–75. 10.1016/j.bbr.2012.12.031 23274842

[87] Gebodh N Vanegas MI Kelly SP. Effects of stimulus size and contrast on the initial primary visual cortical response in humans. Brain Topogr. (2017) 30:450–60. 10.1007/s10548-016-0530-2 28474167 PMC5508105

[88] Wang JB Zheng LJ Cao QJ Wang YF Sun L Zang YF. Inconsistency in abnormal brain activity across cohorts of ADHD-200 in children with attention deficit hyperactivity disorder. Front Neurosci. (2017) 11:320. 10.3389/fnins.2017.00320 28634439 PMC5459906

[89] McLaughlin KA Sheridan MA Winter W Fox NA Zeanah CH Nelson CA. Widespread reductions in cortical thickness following severe early-life deprivation: a neurodevelopmental pathway to attention-deficit/hyperactivity disorder. Biol Psychiatry. (2014) 76:629–38. 10.1016/j.biopsych.2013.08.016 24090797 PMC3969891

[90] Jiang LW Qian RB Fu XM Zhang D Peng N Niu CS. Altered attention networks and DMN in refractory epilepsy: a resting-state functional and causal connectivity study. Epilepsy Behav. (2018) 88:81–6. 10.1016/j.yebeh.2018.06.045 30243110

[91] Sethi A Evelyn-Rahr E Dowell N Jain S Voon V Critchley HD. Magnetization transfer imaging identifies basal ganglia abnormalities in adult ADHD that are invisible to conventional T1 weighted voxel-based morphometry. NeuroImage Clin. (2017) 15:8–14. 10.1016/j.nicl.2017.03.012 28458999 PMC5397127

[92] Zhou M Yang C Bu X Liang Y Lin H Hu X. Abnormal functional network centrality in drug-naive boys with attention-deficit/hyperactivity disorder. Euro Child Adoles Psychiatry. (2019). 10.1007/s00787-019-01297-6. [Epub ahead of print]. 30798413

[93] Wang M Zheng H Du X Dong G. Mapping internet gaming disorder using effective connectivity: a spectral dynamic causal modeling study. Addict Behav. (2019) 90:62–70. 10.1016/j.addbeh.2018.10.019 30366150

[94] Yuan K Cheng P Dong T Bi Y Xing L Yu D. Cortical thickness abnormalities in late adolescence with online gaming addiction. PLoS ONE. (2013) 8:e53055. 10.1371/journal.pone.0053055 23326379 PMC3541375

[95] Yen JY Yen CF Chen CS Tang TC Ko CH. The association between adult ADHD symptoms and internet addiction among college students: the gender difference. Cyberpsychol Behav. (2009) 12:187–91. 10.1089/cpb.2008.0113 19072077

[96] Barber AD Jacobson LA Wexler JL Nebel MB Caffo BS Pekar JJ. Connectivity supporting attention in children with attention deficit hyperactivity disorder. Neuroimage Clin. (2015) 7:68–81. 10.1016/j.nicl.2014.11.011 25610768 PMC4299959

[97] Qian X Castellanos FX Uddin LQ Loo BRY Liu SW Koh HL. Large-scale brain functional network topology disruptions underlie symptom heterogeneity in children with attention-deficit/hyperactivity disorder. NeuroImage Clin. (2019) 21:8. 10.1016/j.nicl.2018.11.010 30472167 PMC6411599

[98] Xia SG Foxe JJ Sroubek AE Branch C Li XB. Topological organization of the small-world visual attention network in children with attention deficit/hyperactivity disorder (ADHD). Front Hum Neurosci. (2014) 8:14. 10.3389/fnhum.2014.00162 24688465 PMC3960496

[99] Tomasi D Volkow ND. Functional connectivity hubs in the human brain. Neuroimage. (2011) 57:908–17. 10.1016/j.neuroimage.2011.05.024 21609769 PMC3129362

